# Buffalo Academy of Medicine

**Published:** 1901-06

**Authors:** George F. Cott


### Society Proceedings.

### BUFFALO ACADEMY OF MEDICINE

*Section on Ophthalmology, Otology, Rhinology and Laryngology, March 18, 1901.*

REPORTED BY GEORGE F. COTT, M. D., Secretary.

THE seventh meeting of this section was held on Monday, March 18, 1901. Dr. F. WHITEHILL HINKEL in the chair Dr. GEORGE F. COTT, made the following report:

### EDEMA OF UVULA.

A recent article in the daily papers which was no doubt read by many physicians has made a wrong impression. The patient, who was aged 50, and under the care of Dr. Chapin, was suffering from chronic Bright's. He was breathing with difficulty. I found great edema of the uvula, which caused loud breathing. The throat was extremely narrow and there was no doubt some edema of the glottis. Heart was very weak and rapid. The patient weighed over 200 pounds. He walked to a couch in another room, where a few drops of a 4 per cent. solution of cocaine were injected under the skin and tracheotomy begun. Just before the trachea was opened, the patient stopped breathing. The windpipe was quickly opened and artificial respiration carried on for three-quarters of an hour, while the patient was put in an inclined plane. Breathing then was again established, but the patient remained quite black and finally died. The cause of death was simply the heart giving out and not due to an operation for quinsy, as has been stated.

Dr. Elmer Starr reported a case of

### ANTRAL DISEASE WITH COMPLICATION.

A suppurating antrum had been drained by extracting a tooth and drilling through the upper jaw. The drainage canal was kept open by passing a probe daily; but eventually the pain accompanying this procedure became so great that the patient frequently allowed two or three days to pass without doing it. One morning, after three days had elapsed without the probe having been used, pain from the pent up matter was so great that in desperation the patient took the probe and attempted to introduce it, but the canal was so nearly closed that considerable force was necessary to overcome the resistance. Suddenly the resistance yielded and the probe entered the antrum, and at the same instant, accompanied by a cracking noise, the eyeball protruded between the eyelids, being pushed completely out of its socket. I suppose the antrum was filled with gas under considerable pressure, and that the probe, when it entered the antrum was pushed with force enough to break the floor of the eye. With the aid of a little pressure the eyeball was soon returned to the socket and suffered no permanent injury from the accident.

Mr. P. H. Meyer then read a paper on

### THE MAKING AND FITTING OF SPECTACLES AND EYE-GLASSES FROM THE OPTICIAN'S STANDPOINT. (See p. 803.)

### DISCUSSION.

Dr. Elmer Starr.—I am glad to have heard Mr. Meyer's paper. We all as medical men can learn something from the optician in the fitting of frames. A dozen or more years ago our patients here were fitted with frames taken from the stock, with little regard for the interpupillary distance, length or curve of templits, or angle at which the lenses were placed. Frequently lenses were decentered in setting them. Now all this is different, thanks to our better opticians, who are doing as good work, I think, as can be found in any part of our country.

All oculists should recognize the importance of correct positions of glasses, and I am glad we have the help of the opticians in this matter. While they are giving us excellent work, I hope they will not be content to stop at this stage, but will soon give us toric and achromatic lenses at less price than they now demand. I have often wished that they might give us more ideal bifocal glasses, in the way of correct tilt or angle of both distance and reading segment.

I wish to thank Mr. Meyer for bringing this important subject before the society.

Dr. A. G. Bennett.—I wish also to thank Mr. Meyer. The oculist and optician have many things in common. The optician has learned many things from the oculist, and the oculist is indebted to the optician for the successful carrying out of his ideas. The dispensing optician holds a very honorable position in society. Of course it is a trade, not a profession, still the dispensing optician is in a position to give sound advice to that class of patients who think that failing vision can always be overcome by the aid of glasses, and by referring such people to a competent oculist may often prevent blindness. I can recall a case of a woman who had glasses adjusted by a quack optician, who, at the time she was fitted was suffering from glaucoma; on the assurance of the optician that it would take some little time to get accustomed to the glasses, she allowed some months to pass before seeking medical aid, by which time vision had almost gone and it required constant attention and energetic treatment to regain even part of the lost vision, and the best obtainable result was only 5-12ths and a very contracted field.

Another case I recall in which a patient suffering from tobacco amblyopia with central scotoma, loss of color sense, and vision reduced to 5-45ths was given a pair of glasses and assured that that was all the treatment he required. In the hands of Mr. Meyer or some other reputable optician, he would not have been given glasses, but would have been advised to seek an oculist's aid. All oculists should support the dispensing optician, as they are a power for good of the country at large.

Dr. FRANK WHITEHILL HINKEL.—I speak from the standpoint neither of the oculist nor of the optician, but of the patient. I know from personal experience how trifling a defect in the lens may affect the wearer. I have had lenses ground for my own wear, that apparently neutralised well, but in which I was conscious of discomfort. My first glasses were made and fitted for me in Philadelphia about twenty years ago by the father of Mr. Fox, who is with us this evening. The grinding and fitting was done then more accurately in Philadelphia than anywhere else, and the mechanical skill and ingenuity of the elder Fox had much to do with this enviable reputation of Philadelphia. I can contrast with much pleasure the entire absence of skilful lens-making in Buffalo, when I first came here seventeen years ago, with the high proficiency of that art here today.

Mr. Fox, optician.—It would aid the optician and patient, if the prescription was given to the patient with the advice to use gold frames rather than filled ones, since the latter do not hold the shape, but bend easily. It is, however, sometimes difficult to make a patient believe it. Filled frames are absolutely no good. But words are necessary on the part of the oculist to make the work much easier for the optician.

Mr. Jarvis, optician.—My mind wanders back to Benjamin Franklin, the inventor of the bifocal lens, the remarkable thing to see through a half lens, an upper and lower for reading, which is now greatly improved. I would like to have heard of methods of proposing lens and mounting.

Dr. MARSHALL CLINTON then read a paper on

FRACTURE OF THE NOSE. (See p. 811.) ok

DISCUSSION.

Dr. EDMOND E. BLAAUW.—I think the subject is so limited in experience without fracture of the lower jaw. Dr. Clinton was fortunate to see a few cases. Fractures of the nasal bones are more important than fractures of the nose. We may divide these fractures into two parts. First, the third portion of the nasal bone is fractured and a part of the superior maxillary bone is broken off, and the cartilage of nose is dislocated backward. Other cases are more direct, up higher, and more simple and more serious. The great problem is to dress as soon as possible and thereby prevent the patient from getting shock. It should be dressed within few hours after accident with anesthetic. To dress the cartilage of the septum take little finger and push the septum against opposite side until the shape of the nose comes back. Dr. Clinton's case is very interesting to the ophthalmologist, as stenosis may arise from the nasal bones pressing upon the lachrymal canal and producing obliteration of the nasal duct.

It is an interesting subject that Dr. Clinton has brought before us tonight, chiefly on account of its rarity. Fractures of nasal bones are as rare as those of the under jaw, that is about 1 per cent. of all the fractures (G. von Bergmann). Fractures of the alæ cartilaginæ naris are of hardly any importance. Fractures of the nose are divided into (*a*) those of the under third portion of the nasal bones, often combined with fractures of the supramax. (proc. frontalis) and nearly always with dislocation, bending or fracture of the cartilaginous septum, and (*b*) those of the upper part of the nasal bones, which must be divided into those where a complication of the brain and its adnexa does and does not apply.

Dr. MARSHALL CLINTON, in closing, said: Dr. Blaauw's figures of 1 per cent. of cases of nasal fractures is too low. This seems a more common injury than he would have us believe.

Adjourned. Fellows present, 22.

---

*Section on Medicine, April 9, 1901.*

REPORTED BY JULIUS ULLMAN, M. D., Secretary.

The chairman, Dr. ELI H. LONG, called the meeting to order at 8.45 p.m., when Dr. A. W. BAYLISS read a paper, entitled,

HOW ARE WE EDUCATING OUR CHILDREN?

This is an age of education, said Dr. Bayliss, but education seems to be extended in only one way. Not enough attention is paid to the physical development of the children in our schools. It is not necessary to make each one of them an athlete, but physical exercise is one way of developing manhood, and, perhaps, I may say, womanhood, if that be not carrying it too far. Education in the schools should be obligatory. We should increase the hours spent by the children in the schools and add to the system of education a course of physical instruction. The time should be divided differently and enough time given to the physical development of the pupils. It seems that physical exercise is compulsory in some of the schools, but not enough time is given to it. I may say, too, that, perhaps, many of the teachers are not sufficiently qualified to instruct in physical culture.

Would it not be well to have inspectors in the schools, to examine the children and decide what form of exercise would be most beneficial to them to take? Physical instructors may be well enough to teach the children, but I am afraid they might overdo it in their enthusiasm. I would advise the appointment of physicians properly qualified to say what exercise is best for each pupil.

The author was inclined to think that children in many cases are unnecessarily hastened through the schools. Instead of the present process of grinding he thought more time should be spent in teaching the children how to study; that more time should be spent in teaching them how to work. Then after they have learned how to study it would be proper for teachers to see that pupils did study. Dr. Bayliss also contended that in his opinion it would be better if the present system of marking the standing of pupils in the schools were abolished; also the honor rolls. It would be better he said if a private record of the standing of each pupil were kept and when a pupil was found to be backward in his or her studies to call the attention of the parents of the child to that fact instead of holding up such a pupil to the ridicule of the school and the neighborhood.

For his part, Dr. Bayliss said he could see no vital reason for the existence of the board of regents, and he gave his reasons. He thought it would be an improvement if the term examinations were abolished and a final examination substituted.

Mr. C. M. Millard, supervisor of the grammar grades, in opening the discussion, said that if anything tangible in the way of improvement was brought out during the discussion the educational persons present would be glad to inform Superintendent Emerson of it. Mr. Millard then told of the work that is being done in the public schools of Buffalo and commented upon some of the suggestions made by Dr. Bayliss. He said it is apparent to everyone that the time spent in mental education and physical education was all out of proportion. At one time it was thought the time spent by the children in play when not in school would be sufficient physical exercise. Some time ago, when physical culture was introduced into the schools, neither the pupils nor the teachers seemed to take much interest in it, but lately all that has changed. He complimented Miss Wiggins on the work she has done in arousing interest in the matter. In the present school hours there is not much time for physical instruction. The hours might be increased. If a physician-inspector were appointed for every school it would cost much money. With the present condition of the city's finances, such a system is not likely to be established for some time.

Mr. Millard suggested as another plan that it might be well to give four or five more assistants to the present physical instructors. He asked Dr. Bayliss to outline his suggestion for the introduction of physicians to examine the children so as to determine what course of physical instruction would be best for them to pursue. Dr. Bayliss said his plan was to have a physician at the head of the physical-instruction system and to have a corps of inspectors work under him and follow his instructions.

The teachers of today, Mr. Millard afterward argued, are trying to teach the pupils how to study. He saw good in the board of regents.

Miss Alta J. Wiggins, director of physical culture in the school department, said the work of giving the children physical instruction was important, but practically little is being done in this big field. There is hope that more can be accomplished. There are all sorts of physical effects that retard a child's progress in school, and it would be a good plan to have physicians to whom such cases could be referred. We are not trying to make athletes out of the pupils, but are working to counteract the influence of school study. Bad ventilation is the cause of much trouble. Then, again, the desks—those that are not adjustable desks—are an abomination. They are not suited to the correct sitting posture of a child. A child cannot be comfortable in them, but we are trying to get the children to sit in the proper way in spite of the construction of the desks. Miss Wiggins said the adjustable desks—those that are all right—are vastly in the minority.

Dr. P. W. VanPeyma, chairman of the board of school examiners, followed with an argument on the importance of proper ventilation of the school rooms. The children cannot have good health if they do not have fresh air. It is important also that the rooms be properly lighted. Dr. Van Peyma related an instance where the plans of an architect for an addition to one of the schools made it necessary for the children to sit with their eyes facing the light, which would have been injurious to them. He was surprised at the sweeping statement of Miss Wiggins in reference to the school desks that are not of the kind called adjustable desks. Speaking on physical instruction, he told of the schools of Paris, where, he said, each group of schools has its building especially for use as a gymnasium. Buffalo should have something similar.

Dr. Henry R. Hopkins, professor of hygiene, University of Buffalo, said: Physical education is more important to a child than the knowledge of how many rivers empty into the Caspian sea. Too much work is crowded into the present school hours. If two-thirds of it were taken away it would be immensely to the advantage of the pupils. Public playgrounds, public baths and gymnasiums would be good for the children. Exercise out of school would diminish the extraordinary artificial life of the children who cannot leave the crowded houses for the street without being run over. It is not proper that there should be children in the schools who are handicapped by defects that cannot be remedied. Speaking of the ventilation of buildings, Dr. Hopkins suggested that it would be a good thing if the states were to license architects; the state should know something about the intelligence of men in that profession.

Adolph Mier, instructor in the Buffalo gymnasium, followed with an argument showing the importance of physical exercise and of the benefits that eventually result to those who indulge in it. It would be one of the best things in the world for the school children, he said, and its importance should make it obligatory.

The section then adjourned.

---

*Section on Obstetrics and Gynecology, April 23, 1901.*

Reported by WILLIAM G. RING, M. D., Secretary.

The regular monthly meeting of this section was held April 23, 1901, at 8.30 p.m., Dr. A. J. Colton presiding.

The minutes of the previous meeting were read and approved.

Dr. M. D. Mann read a paper on

THE OPERATIVE TREATMENT OF PROLAPSUS UTERI.

DISCUSSION.

Dr. H. E. Hayd approved of the method advocated by Dr. Mann, but made further suggestions as to the operative treatment of extreme cases of prolapse. Ventral fixation, next to an Alexander operation, has given me great satisfaction, especially in women who have passed beyond the child-bearing period. Every case must be considered from the point of its pathology.

Dr. J. W. Grosvenor.—There is a certain class of cases of prolapse, that is in aged women, which are extremely difficult to treat. Cited two cases in which the prolapse amounted to procidentia. Had tried sponges, India rubber balls, metallic cup and stem, cotton pledgets, all of which were unsuccessful. He would like to ask Dr. Mann what he would do in cases of aged women?

Dr. H. D. INGRAHAM.—It is a more serious condition than Dr. Mann has inferred. I have had many cases and many failures. In women who are pretty well advanced, someone, I do not know who, has suggested putting in two silver wires, through the neck of the uterus (Machinrodt's operation). Denudation of vagina; construction of same, failed. Recommended amputation of the cervix uteri. We should not shorten the posterior wall of vagina. He would not recommend Alexander's operation when women are well along toward menopause, but would use ventral fixation.

Dr. P. W. VAN PEYMA.—In answer to Dr. Grosvenor, I have had some cases in old women. I use cup and stem pessary, with belt and rubber strips externally (McIntosh pessary). Dr. Carl Braun says ventral fixation is a failure.

Dr. M. D. MANN, in closing, and remarking in regard to cases cited by Dr. Grosvenor, related method used in the South during slave days by hammock and white oak bark. Had successfully used Thomas's modification of Cutler's pessary, and it should be used more. He thought the McIntosh pessary an abomination. You cannot do Alexander's operation in moderate cases; it is only part of what should be done—it should be supplemented by Pryor's operation. I do not believe in taking out the uterus in procidentia. The condition is almost an incurable one. I have had poor results in shortening the utero-sacral ligaments. I much prefer Pryor's operation.

The second paper of the evening was read by Dr. J. V. WOODRUFF on

THE CORRECTION OF FACE PRESENTATION.

DISCUSSION.

Dr. P. W. VAN PEYMA.—Many years ago Dr. Hauenstein delivered in the knee chest position. He was much interested in a paper of Dr. Banta's. Cannot say Dr. Woodruff's paper does not appeal to me very much, but advocates deep anesthesia. Any case indicating bringing down the occiput can be done under deep anesthesia. Reynolds's classification cleared up many points in my mind; he divides all cases into four classes, which are treated accordingly.

Dr. LUDWIG SCHROETER.—It seems to me we should make a distinction between cases of normal and flat pelvis. If a normal pelvis, leave it alone and wait. I prefer podalic version for changing the presentation. In impeded cases there is danger of rupture of the uterus. Perforate in eclamptic cases where you cannot wait.

Dr. J. W. Grosvenor asked if abdominal section was not preferable to perforation?

Dr. John Hauenstein.—I came to hear, not to be heard. It is six years since I practised midwifery. I gave in an address, twenty years ago, my method of procedure. I made cephalic version as soon as the knee-chest position was adopted in this country. Two years ago I explained my plan of cephalic version before this society. I have no cause to change my opinion. Position is one of the most important points in making version without pain and inconvenience to the parturient. I never found it necessary to give chloroform or ether. The head is apt to slip; this can be overcome by making indentation of scalp, and thus bring the vertex down.

Dr. H. D. Ingraham.—I have seen two or three cases, but left them alone. I saw Dr. Banta make the change easily. That is my experience.

Dr. M. D. Mann.—All my cases were consultation cases. I believe in replacing the head. I remember a case with Dr. Diehl; it slipped back a dozen times. We made podalic version. It was due to muscular spasm. Another case with Dr. Smith. We got Tarnier forceps in and delivered. Mento-posterior position of head is an impossible position for delivery; it must be replaced. Cesarean section or symphysiotomy could be done. In Dr. Reynolds's third position, let it alone. In the Trendelenburg, we have a valuable position for several conditions. It has great advantages over the knee-chest or perpendicular positions. Deep anesthesia with position is of great advantage.

Dr. Eli H. Long expressed pleasure of hearing paper, and cited his experience of one face case, and three brow cases; all changed to vertex positions and terminated favorably by forceps delivery. Relies on knee-chest position and version.

Dr. Woodruff, in closing, stated that he had used profound anesthesia but failed. Dr. Mann's suggestion of Trendelenburg position on a chair is a good one, but think if good the perpendicular position must be. Have never been to a birth case where there was not any quantity of help. Posture was of great value in the case related by me. Would prefer to perforate instead of a Cesarean section.

Adjourned at 10.25. Present, 25.

